# Cantharidin Impedes Activity of Glutathione *S*-Transferase in the Midgut of *Helicoverpa armigera* Hübner

**DOI:** 10.3390/ijms14035482

**Published:** 2013-03-08

**Authors:** Rashid Ahmed Khan, Ji Yuan Liu, Maryam Rashid, Dun Wang, Ya Lin Zhang

**Affiliations:** 1 Key Laboratory of Plant Protection Resources and Pest Management, Ministry of Education, College of Plant Protection, Northwest A&F University, Yangling 712100, Shaanxi, China; E-Mails: rashidpp2004@yahoo.co.uk (R.A.K.); liujiyuan@nwsuaf.edu.cn (J.Y.L.); smilejust9@yahoo.com (M.R.); 2 Institute of Entomology, Northwest A&F University, Yangling 712100, Shaanxi, China

**Keywords:** glutathione *S*-transferases, *Helicoverpa armigera*, cantharidin, mRNA, molecular docking simulations

## Abstract

Previous investigations have implicated glutathione *S*-transferases (GSTs) as one of the major reasons for insecticide resistance. Therefore, effectiveness of new candidate compounds depends on their ability to inhibit GSTs to prevent metabolic detoxification by insects. Cantharidin, a terpenoid compound of insect origin, has been developed as a bio-pesticide in China, and proves highly toxic to a wide range of insects, especially lepidopteran. In the present study, we test cantharidin as a model compound for its toxicity, effects on the mRNA transcription of a model *Helicoverpa armigera* glutathione *S*-transferase gene (HaGST) and also for its putative inhibitory effect on the catalytic activity of GSTs, both *in vivo* and *in vitro* in *Helicoverpa armigera*, employing molecular and biochemical methods. Bioassay results showed that cantharidin was highly toxic to *H. armigera*. Real-time qPCR showed down-regulation of the HaGST at the mRNA transcript ranging from 2.5 to 12.5 folds while biochemical assays showed *in vivo* inhibition of GSTs in midgut and *in vitro* inhibition of rHaGST. Binding of cantharidin to HaGST was rationalized by homology and molecular docking simulations using a model GST (1PN9) as a template structure. Molecular docking simulations also confirmed accurate docking of the cantharidin molecule to the active site of HaGST impeding its catalytic activity.

## 1. Introduction

Glutathione *S*-transferase (GST EC 2.5.1.18) is an important family of multifunctional isozymes found in all eukaryotes. One of the main metabolic functions of GST is to catalyse xenobiotics, including pesticides in the mercapturic acid pathway leading to the elimination of toxic compounds [1]. GSTs also convert a reactive lipophilic molecule into a water-soluble, non-reactive conjugate which may easily be excreted [2]. This family of enzymes has been implicated as one of the major mechanisms for neutralizing the toxic effects of insecticides in insects [3,4].

In recent years, the management of *Helicoverpa armigera*, the American bollworm, has become increasingly difficult due to the development of resistance to various groups of insecticides, particularly pyrethroids and cyclodyienes [5]. *H. armigera* is an important polyphagous pest of cotton and many other crops of agricultural importance all over the world. In insects, GSTs provide an important defense mechanism against plant allelochemicals [6] as well as insecticides [7]. An increased level of GSTs has been regarded as causing organochlorine and organophosphorus insecticide resistance [8–14].

The active constituent of mylabris, cantharidin is produced by as many as 1500 different species of blister beetles, with the Spanish fly *Cantharis vesicatoria* probably being the best known source [15,16]. Cantharidin was first isolated by Robiquet, a French chemist in 1810. It has an important role in the ecology of different kinds of insects that use or produce it as a defense to preserve their eggs from predators [17].

Recently this defensive tool has been developed into a bio-pesticide and one of the formulations as an emulsifiable concentrate (EC) has been registered for the control of lepidopteran pests while other formulations of cantharidin and its analogues are under field trial for registration. Cantharidin as 1.0% EC has been tested for its environmental safety and toxicity was found to be within safe limits for ladybugs, quail and soil microorganisms [18]. The antifeedant activity of cantharidin and its toxicity was earlier established against armyworm, *Mythimna separata*[19]. Furthermore, inhibitory effect of cantharidin on GSTs was reported in *M. separata*[20]. However, its toxicity and inhibitory effect on GSTs in *H. armigera* have not yet been investigated in detail.

At present there is very little information about the toxicity mechanism of cantharidin to insects and its interaction with metabolizing enzymes, especially GSTs. In the present study, our aim was to investigate the effect of cantharidin on mRNA transcript of HaGST (GenBank accession no EF033109) and also its putative role in the inhibition of GSTs in *H. armigera* both *in vivo* and *in vitro*. For this purpose, we cloned a model *H. armigera* GST gene (HaGST) which was previously reported for its role in insect defense mechanism [21] and prokaryotically expressed it to get soluble recombinant protein, rHaGST. Homology modeling and molecular docking simulation techniques were employed to rationalize our experimental results.

## 2. Results and Discussion

### 2.1. Bioassay

**Figure 1:**
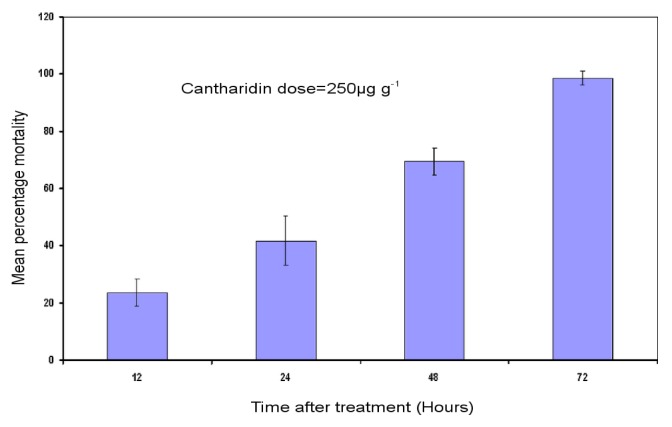
Mean percentage mortalities of *H. armigera*. Third-instar larvae were subjected to bioassay. Error bars show ± SD among three replications. Number of third-instar larvae tested per treatment (*n* = 72).

Bioassay results showed an increase in levels of *H. armigera* mortality with time after feeding on cantharidin-treated artificial diet. The artificial diet incorporated cantharidin of 250 μg g^−1^ at 12, 24, 48, 72 h after treatment caused significant larval mortality of 23%, 41% and 69% and 98%, respectively (Figure 1).

### 2.2. SDS-PAGE Analysis

**Figure 2:**
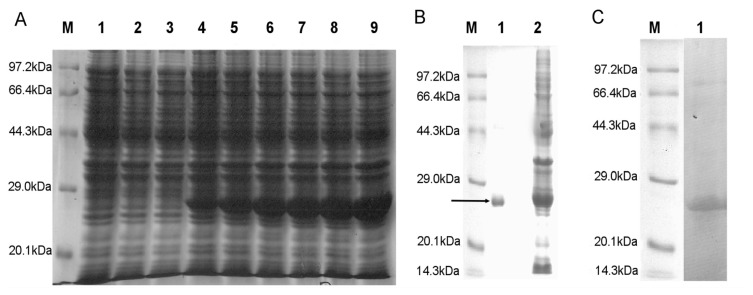
SDS-PAGE analysis of fusion protein. (**A**) Lane M, Protein weight marker; Lane1, DE-3; Lane 2, DE-3 + pET28a; Lane 3, DE-3 + pET28a-HaGST (without IPTG); Lane 4–6, expression level of the soluble fusion protein at 1–6 h; (**B**) Purified soluble rHaGST. Lane M, Protein weight marker; Lane 1, Purified rHaGST by Ni^2+^-nitrilotriacetate (NTA) column; Lane 2, non purified protein; (**C**) Immunoblotting of rHaGST with 6× His mouse monoclonal primary antibody. Lane M, Molecular weight marker; Lane 1, rHaGST detected by peroxidase conjugated goat anti-mouse IgG secondary antibody.

The positive clones were transformed in BL-21 (DE-3) and protein expression induced by the addition of IPTG was detected initially by SDS-PAGE using standard protein molecular weight marker. The expected band was detected at 27 kDa as the MW of the HaGST is about 24 kDa and the pET-28a tag is about 3 kDa (Figure 2). The expression of recombinant protein was detected by 6× His mouse monoclonal primary antibody on a PVDF membrane.

### 2.3. Specific Activity of HaGST

**Figure 3:**
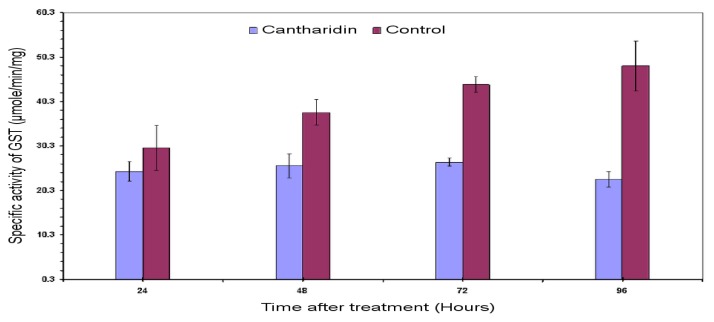
Specific activity of the GSTs in larval midgut of *H. armigera* subjected to sub-lethal dose of cantharidin using glutathione (GSH) and 1-chloro-2,4-dinitrobenzene (CDNB) secondary substrate. The activity of the GSTs was measured at 340 nm both in treated and control samples.

The inhibitory effects of cantharidin *in vivo* on GSTs enzyme within midguts dissected from larvae treated with sub-lethal dose of cantharidin is shown in Figure 3. A sub-lethal dose of 25 μg g^−1^ exerted inhibitory effects on the GSTs in midgut as compared to the untreated control. Data showed that the specific activity of the GSTs tended to decrease in treatment from 24 to 96 h, whereas specific activity tended to increase in untreated controls. The inhibitory effect on the activity at 96 h after treatment was 22.8 compared to 48.32 μM min^−1^ mg^−1^ of control.

### 2.4. Kinetic Properties of GSTs

**Figure 4:**
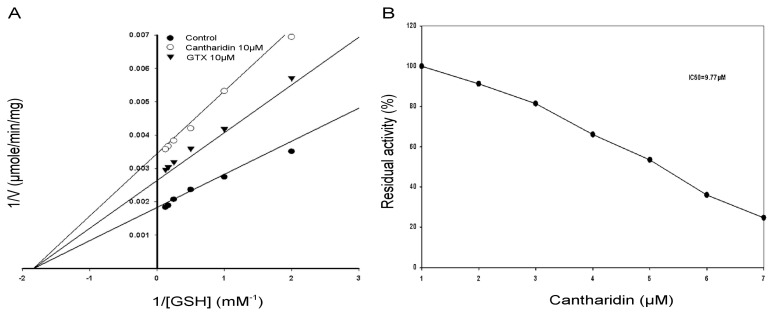
Lineweaver-Burk plot of the GSTs activity in crude enzyme extract. (**A**) Specific activity of GSTs with and without cantharidin. IC50 value of cantharidin for the GSTs using glutathione (GSH) and 1-chloro-2,4-dinitrobenzene (CDNB) secondary substrate; (**B**) IC50 value was obtained using a plot of percent activities *vs.* varying concentrations of cantharidin. *S*-Hexylglutathione (GTX) was used as positive control.

The inhibitory effects of cantharidin on the GSTs enzyme extract activity using GSH as substrate is shown in Figure 4. To find out the kind of cantharidin inhibition of the GSTs enzyme extract by cantharidin, the GSTs activity was calculated with variable concentrations of GSH. A Lineweaver-Burk plot with GSH as the variable substrate and for the type of inhibition by cantharidin is shown in Figure 4A. The Lineweaver-Burk plot revealed that cantharidin inhibited GSTs non-competitively as *V*_max_ lowered down, whereas Km remained unchanged with respect to GSH.

### 2.5. Kinetic Properties of Purified rHaGST

**Figure 5:**
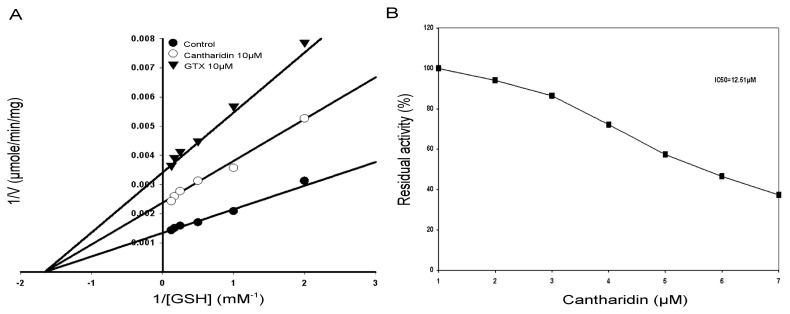
Lineweaver-Burk plot of the purified soluble rHaGST. (**A**) Specific activity of GSTs with and without cantharidin. IC50 value of cantharidin for GSTs using glutathione (GSH) and 1-chloro-2,4-dinitrobenzene (CDNB) secondary substrate; (**B**) IC50 value was obtained using a plot of percent activities *vs.* varying concentrations of cantharidin. *S*-Hexylglutathione (GTX) was used as positive control.

The inhibitory effects of the cantharidin on the affinity of the column purified soluble rHaGST activity using GSH as substrate is shown in Figure 5. To determine the type of inhibition of the rHaGST by cantharidin, the rHaGST activity was calculated under different concentrations of GSH. The Lineweaver-Burk plot for GSH as the variable substrate and type of inhibition is shown in Figure 5A. The Lineweaver-Burk plot revealed that cantharidin inhibited rHaGST non-competitively as *V*_max_ lowered, whereas Km remained unchanged with respect to GSH.

### 2.6. IC50 of Cantharidin

To calculate IC50 of cantharidin for the GSTs and the soluble purified rHaGST, variable concentrations of cantharidin were used using GSH as substrate. Results showed that cantharidin inhibited activity of the GSTs and the purified rHaGST in a dose-dependent manner with 50% inhibitory concentration at 9.77 μM (Figure 4B) and 12.5 μM (Figure 5B), respectively.

### 2.7. Time Course Expression Profile of the HaGST Gene by Real-Time qPCR

**Figure 6:**
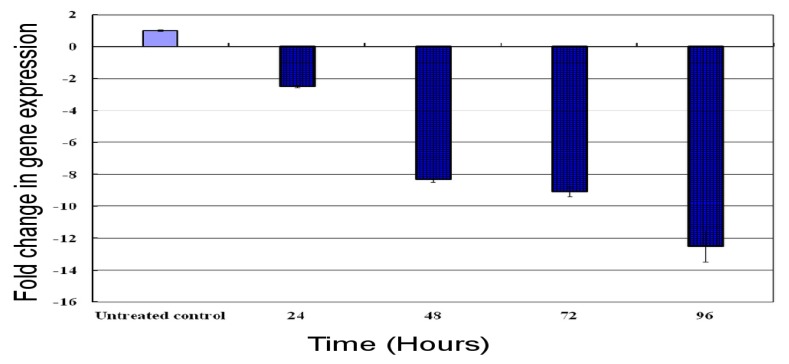
The real-time q-PCR analysis of the HaGST mRNA transcript at different time intervals from 24 to 96 h. Target gene expression was normalized by comparing ΔΔCT to an untreated control. Error bars show standard deviation using three replications.

Real-time qPCR was carried out to investigate the effect of cantharidin on the mRNA transcript of the HaGST based on time after treatment. Results showed that the HaGST was down-regulated at 24, 48, 72 and 96 h after treatment. Normalized expression results revealed that the HaGST was down regulated by 2.5, 8.3, 9.09 and 12.5 folds after 24, 48, 72 and 96 h, respectively (Figure 6).

### 2.8. Homology Modeling of HaGST

**Figure 7:**
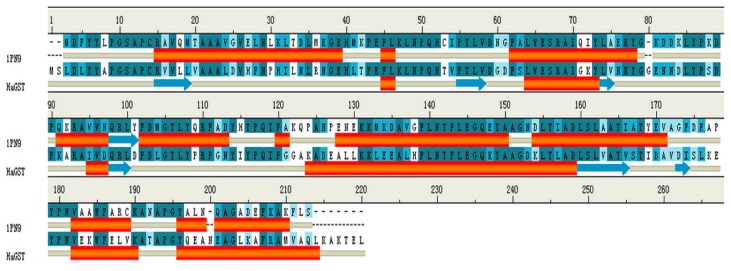
Sequence alignment results. The target protein HaGST and template protein, IPN9. Deep green color indicates conserved residues in both the sequences. Red bands show α helices while blue arrows show β sheets.

**Figure 8:**
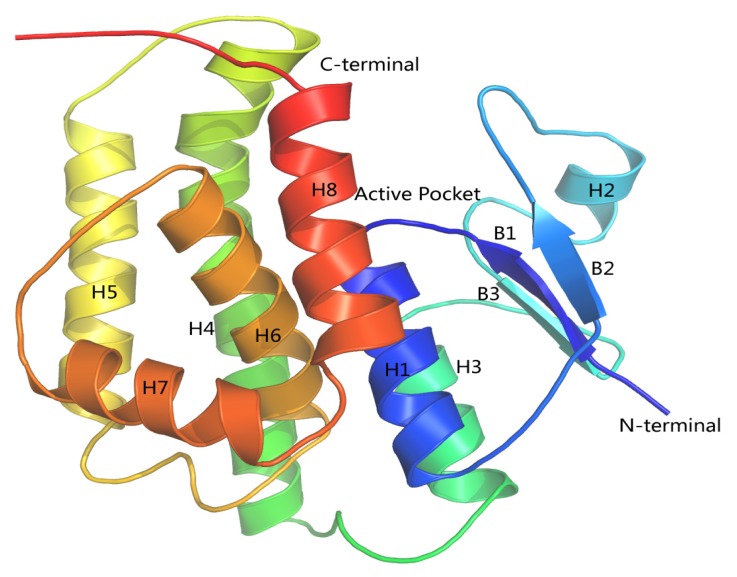
The ribbon representation of the 3-D model of the HaGST. Blue and red colors represent a chain trace from the *N*-terminus to *C*-terminus, respectively. α helices and β sheets are represented by H and B, respectively. The active site is located in the cleft formed at the interface of H8, H3 and between loops of H2, B3.

In order to study the binding of cantharidin with HaGST, a better crystallographic *R*-factor (20.9%) and higher overall sequence identity (57%) was considered (Figure 7). We finally selected the complete crystal structure of an insect delta-class glutathione *S*-transferase from a DDT-resistant strain of the malaria vector *Anopheles gambiae* in complex with its inhibitor named *S*-hexylglutathione (GTX) as the template (PDB:1PN9) [22] at a resolution of 2.0 Å. The best model was selected with the lowest value of DOPE assessment score (−27,127.846). The analysis of the Ramachandran plot showed that 97.2% (212/218) of all residues were in favored (98%) regions, and 99.1% (216/218) of all residues were in allowed (>99.8%) regions. Only two amino acids GLU66 and ALA212 were found in the disallowed region of the Ramachandran plot. The resultant 3D structure of the HaGST is shown in Figure 8. The active site is located in a deep cleft formed at the interface of the two domains.

### 2.9. Molecular Docking Simulations

**Figure 9:**
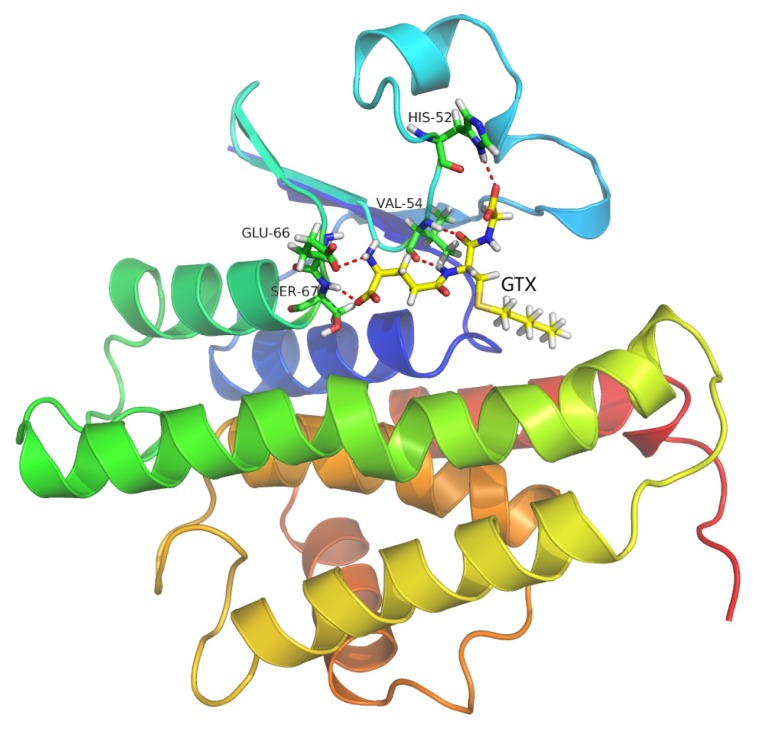
The binding model of GTX with the HaGST. Red dotted lines show hydrogen bonding among the corresponding atoms of amino acid residues of the active site and GTX.

**Figure 10:**
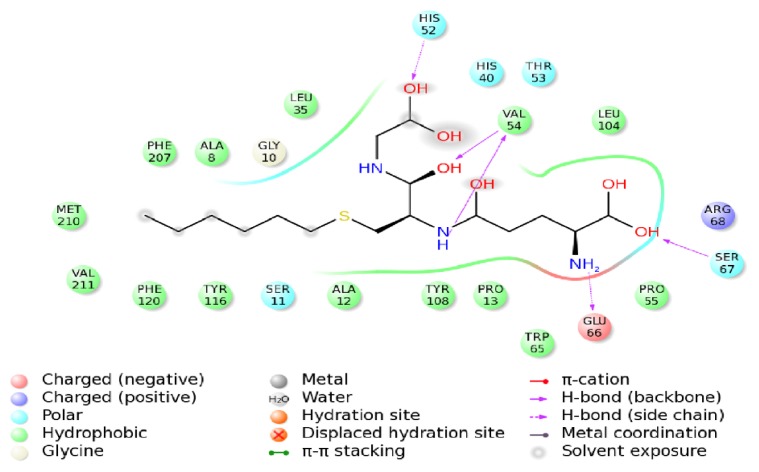
The interaction diagram of the insect delta-class HaGST from *Helicoverpa armigera* with its inhibitor, GTX.

**Figure 11:**
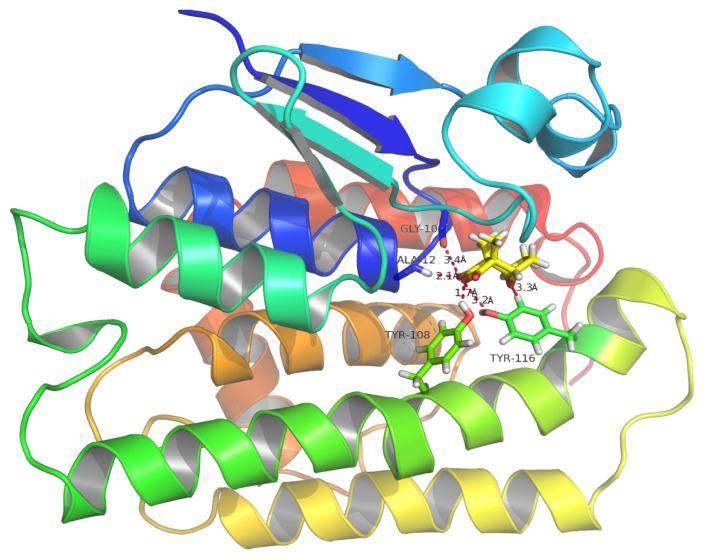
Binding mode of the cantharidin-HaGST complex. Red dotted lines show hydrogen bonding between the amino acid residues of the active site and atoms of cantharidin. The O atom of GLY10 and NH atom of ALA12 interact with the cantharidin O3 atom, simultaneously by hydrogen bonding. The OH atom of TRY116 also interacts with the O3 atom of cantharidin. A OH atom of TRY108 forming a hydrogen bond with O1 of cantharidin plays an important role in binding affinity based on its lowest inter-atomic distance.

As shown in Figure 9 the final binding mode of the GTX-HaGST was obtained by molecular docking and superimposition (RMSD of only 0.210Å) of the conformations of the GTX in the 1PN9 and HaGST. The O26 atom in the GTX and the NH atom of the HIS52 side chain formed hydrogen bonds having a distance of 2.2 Å between atoms. The NH and the O atoms, which are derived from the VAL54 backbone, interact with the O13 atom and the H8 atom in the GTX by hydrogen bonds respectively, having distances of 1.8 Å and 2.0 Å between them. The NH atom of SER67 backbone also interacts with the O5 atom of GTX with a hydrogen bond distance of 1.9 Å between atoms. The hydrogen bonds are formed between the H2 atom of the GTX and the O atom from GLU66 side chain whose distance is 2.4 Å (Figure 10). While comparing the schematic representation of residual-ligand hydrogen bond interactions, only one interaction site in insect delta class GST, ILE52 in *Anopheles gambiae* may have mutated to VAL54 in *Helicoverpa armigera*. Although the VAL54 and the ILE52 are non-polar hydrophobic amino acids, the NH atom derived from the backbone of ILE52 only interacts with the O13 atom of the GTX. Based on our findings, it could be assumed that the hydrogen bond network is more stable in the GTX-HaGST 3D model complex than those in the crystal structure named 1PN9. Based on the docking work mentioned above, the accurate binding mode of the HaGST 3D model with the cantharidin was obtained as shown in Figure 11.

**Figure 12:**
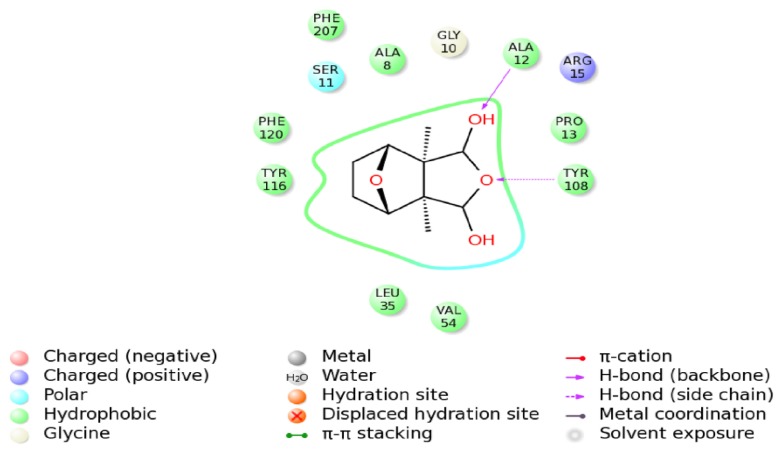
The Interaction diagram of the delta class HaGST with cantharidin. ALA12 and TRY108 are mainly responsible for making hydrogen bonds with the O atoms of cantharidin.

The schematic representation of residue-ligand interactions for cantharidin is shown in Figure 12. The cantharidin docks inside the active site formed by amino acid residues TYR116, PHE120, SER11, ALA8, GLY10, ALA12, ARG15, PRO13, TYR108, LEU35, VAL54. The O atom of GLY10 and the NH atom of ALA12 which are both derived from the backbone of the HaGST 3D model simultaneously interact with the O3 atom of the cantharidin by hydrogen bonds, whose distances are 3.4 Å and 2.1 Å, respectively. The OH atom from the side chain of TYR116 also interacts with the O3 atom of the cantharidin by hydrogen bonds. The hydrogen bonds are formed between the OH atom of TYR108 and the O1 atom of the cantharidin which are only1.7 Å apart. The smaller distance of 1.7 Å between the OH atom of TYR108 and the O1 atom of the cantharidin may have a profound impact on their binding affinity. The hydrogen bond network occupying the active site of HaGST allows the formation of the stable complex with cantharidin.

### 2.10. Binding Energy Calculations

As shown in Table 1, the calculated binding energy delta G (DG) indicates that cantharidin has a higher binding affinity than the GTX.

In our current research we have used GST as a molecular target of cantharidin since GSTs plays an important role in defense against plant allelochemicals [6] as well as insecticides [7].

Earlier studies have suggested the increased level of GSTs as one of the major reasons for the development of resistance using metabolic detoxification of insecticides. Many researchers have documented the development of insecticide resistance caused by increased levels of GSTs especially in diamondback moth (DBM) and *H. armigera* which are phylogenetically related. In one report, indiscriminate use of insecticides, multiple generation of DBM per annum and year round availability of host crops have been mentioned as reasons for development of resistance in this pest to all kinds of insecticides [23,24]. There is a clear correlation between resistance and level of GSTs in insects. Likewise, there is higher activity of GST in the DBM-R population by a 3 to 4 fold increase in enzyme activity over susceptible strains of *Plutella xylostella*[25]. Similarly, a 1.5 to 2 fold increase in parathione selected DBM was observed [26]. Higher activity of GSTs in pyrethroid resistant strain has established that GST plays an important role as a detoxifying mechanism in DBM [27]. There are earlier reports of resistance in *H. armigera* to synthetic and nonsynthetic pyrethroid insecticides in China, with resistance to deltamethrin, cyhalothrin, fenpropathrin, esfenvalerate, cyfluthrin and methomyl ranging between 10 and 50-fold [28]. GST could provide passive protection by binding pyrethroid molecules in a sequestration mechanism in resistant field strains of *H. armigera*[29]. Detoxifying GSTs are a family of enzymes that catalyze the conjugation of glutathione with electrophilic substrates including insecticides [30]. The GSTs are involved in *O*-dealkylation or dearylation of OPs [31]. High frequencies of profenofos resistance were moderately correlated with GST activity toward 1-chloro-2,4-dinitrobenzene in larvae of *H. virescens* that were collected in Louisiana cotton fields during the 1995 cotton growing season [32]. A recent study suggests that GSTs act as an antioxidant-defense agent and confer pyrethroid resistance in *Nilaparvata lugens* and possibly in other insects [33]. Enhanced activities of GSTs that confer insecticide resistance result from both quantitative and qualitative alterations in gene expression. First, there is evidence for over-expression of one or more GST isoforms in resistant insects. For example, the highest activity found in an insecticide-resistant strain of *M. domestica* is correlated with a high level of GST1 transcript [12].

Our investigations revealed that cantharidin is an effective inhibitor of the GSTs and the HaGST in the midgut of *H. armigera* with IC50 of 9.77 and 12.5 μM, respectively. The low value of IC50 *in vivo* suggests that it may be a general inhibitor of GSTs. Enzyme kinetics studies were carried out to detect the inhibitory mechanism of GSTs in enzyme extract and soluble purified rHaGST by cantharidin with respect to GSH. Results revealed that cantharidin was non-competitive inhibitor of the GSTs and the HaGST with respect to GSH, suggesting that cantharidin binding to GSTs may have caused conformational changes consequently leading to the enzyme inactivation. Furthermore, cantharidin has the potential to bind to GSTs through hydrogen a bond causing steric obstruction leading to inactivation of catalytic activity.

Homology modeling and molecular docking simulations were employed to rationalize our results more precisely. Our docking results confirmed putative binding of cantharidin to the catalytic active site residues, ALA12 and TRY108, resultantly causing conformational changes that lead to the inactivation of enzyme catalytic activity.

## 3. Experimental Section

### 3.1. Insects

*Helicoverpa armigera* larvae were procured from Henan Jiyuan Baiyun Industry Co., Ltd. China and reared on artificial diet [34] until F1 were available for use in the experiments. Groups of 24 larvae were placed into 24 chamber plastic boxes obtained from the company. The boxes were placed in an incubator at 27 + 1 °C and 40% to 50% RH with a 12 h photoperiod.

### 3.2. Bioassay

Diet incorporation bioassay was used to determine the toxicity of cantharidin. Batches of healthy homogeneous third-instar larvae were selected for bioassay. A homogeneous group comprising 24 larvae per replication was subjected to bioassay. The bioassay experiment was replicated thrice. Cantharidin dissolved in acetone was added to the semisolid artificial diet at the rate of 250 μg g^−1^and mixed well. Acetone was allowed to evaporate for 1 h before allowing insects to feed. The larvae were starved for 8 h before their introduction to treated diet. One larva of third-instar was introduced to each cell of the 24-cell plastic bioassay tray. The mortality data were recorded at 12, 24, 48 and 72 h.

### 3.3. Larval Treatment

Cantharidin used in the experiment was extracted in the laboratory. The third-instar larvae were selected for this study. The insects were starved for 8 h before their introduction to the cantharidin-treated artificial diet containing 25 μg g^−1^ cantharidin. Afterward, larvae were collected at an interval of 24, 48, 72 and 96 h. The collected larvae were flash frozen in liquid nitrogen for storage at −80 °C and subsequently used for extraction of enzyme extract and total RNA for synthesis of cDNAs.

### 3.4. Total RNA Preparation and Synthesis of cDNAs Template for Real-Time qPCR

A total of 10 × 3 larvae per time interval (10 larvae per replication), treated as mentioned above were used for extraction of total RNA. At first midguts were dissected from the larvae stored at −80 °C and homogenized using liquid nitrogen before addition of RNAiso Plus (TaKaRa, Dalian, China). RNA was extracted under the guidelines of the manufacturer’s instructions. The quality of the RNA samples was examined by running on 1% (*w*/*v*) agarose gel. DNA contamination was removed by DNaseI (Fermentas, Beijing, China). The cDNAs were synthesized by reverse transcription using RevertAid™ Reverse Transcriptase (Fermentas, Beijing, China) in a 20 μL reaction containing 5 μL total RNA having 1 μg RNA, oligo (dT)_18_ primer 1 μL, 5× Reaction Buffer 4 μL, 10 mM dNTP Mix 2 μL and RevertAid™ M-MuLV Reverse Transcriptase (200 U μL^−1^) 1 μL. The reaction mixture was incubated for 60 min at 42 °C. The reaction was terminated by heating at 70 °C for 10 min. The product of the reverse transcription reaction was stored at −80 °C.

### 3.5. Cloning of Glutathione *S*-Transferase Gene from *H. armigera*

Glutathione *S*-transferase (GenBank accession no. EF033109) was amplified from cDNA by polymerase chain reaction (PCR) using a pair of sense and antisense primers, respectively (Table 2). The *Bam*HI restriction site was incorporated to sense primer, whereas *Hind*III restriction site was incorporated to antisense primer for double restriction digestion reaction. The amplification reaction was performed by the PCR program: first step denaturation for 3 min at 95 °C followed by 34 cycles of 95 °C for 30 s, 55 °C 30 s, 72 °C for 1 min and final extension of 5 min at 72 °C. The PCR product was run on 1% (*w*/*v*) agarose gel and visualized by ethidium bromide using the BioRad imaging system. Target gene amplified product was gel purified by a gel extraction kit (Biomiga, San Diego, CA, USA). Gel purified PCR product was then ligated to pMD-19T vector (TaKaRa, Dalian, China) and transformed into *Escherichia coli* DH5α. The transformants were selected on LB agar plates containing 50 μg mL^−1^ kanamycin after overnight incubation at 37 °C. The resultant PCR clones were sequenced by Shanghai Sunny Biotech, Shanghai, China.

### 3.6. Construction of Recombinant Expression Plasmid

The pMD19T-HaGST was subjected to double restriction digestion by *Bam*HI and *Hind*III. The digested fragments were gel purified and ligated into the prokaryotic expression vector, pET-28a (Novagen, Darmstadt, Germany) using TaKaRa quick ligation kit to secure the recombinant plasmid, rHaGST. The ligation reaction was transformed into BL-21 (DE-3) competent cells. The transformed Bl-21 cells were cultured in LB media containing kanamycin (50 μg mL^−1^) at 37 °C with 220 rpm shaking until the absorbance at OD_600_ reached 0.5 nm, then isopropyl-β-d-thiogalactopyranoside (IPTG) was added to the culture in final concentration of 1 mM at 30 °C for expression of recombinant protein.

### 3.7. SDS-PAGE Analysis of Recombinant Protein and Immunoblotting

The expressed recombinant protein of 29.81 kDa was confirmed and visualized by 12% SDS-PAGE using a standard protein marker (TaKaRa, Dalian, China). After running the recombinant protein on 12% SDS-PAGE, the gel was subjected to Coomassie brilliant blue R250 staining and proteins were transferred to a polyvinylidene fluoride membrane (PVDF). Immunoblotting was done [35] with 6-His monoclonal primary antibody and peroxidase-conjugated goat anti-mouse IgG secondary antibody.

### 3.8. Prokaryotic Expression and Purification of the Soluble Recombinant Protein, rHaGST

The *E. coli* strain Bl-21 (DE-3) cells with the rHaGST were grown at 37 °C in 50 mL LB media containing 50 μg mL^−1^ kanamycin until the OD_600_ reached 1. The media was then added 0.5 mM IPTG and cells were grown at 28 °C for 6 h with shaking at 200 rpm. The cells were harvested at 10,000 rpm for one min. The resultant pellet was washed with sterile water three times and finally mixed with the binding buffer containing 20 mM imidazol in equilibration buffer. Lysozyme was added to the mixture at the rate of 1 mg mL^−1^. After incubation for 30 min on ice, cells were lysed by gentle vortexing. The lysate was subjected to centrifugation at 12,000 rpm for 10 min to remove the cellular debris. The supernatant was passed through a 0.45 nM syringe filter for removal of any remaining debris. The protein extract obtained was passed through a Ni^2+^-nitrilotriacetate (NTA) chromatographic column. The column was washed with equilibration buffer containing, 300 mM NaCl, 50 mM sodium phosphate buffer, 10 mM imidazol and 0.01 M Tris-Cl (pH 8.0). Protein was eluted with a linear imidazole gradient of 50, 100, 150 and 200 mM. The eluted protein was desalted using a dialysis membrane against 50 mM sodium phosphate buffer, pH 7.2 for 24 h at 4 °C.

### 3.9. Enzyme Extract Preparation

A total of 10 × 3 larvae per time interval (10 larvae per replication), treated as mentioned above were used for enzyme extraction. The midguts dissected from the larvae were washed with 1× phosphate buffer and homogenized in 0.1 M potassium phosphate buffer, pH 6.5 on ice using a glass homogenizer. The homogenates were centrifuged at 10,000*g* for 15 min at 4 °C. The supernatants were used as an enzyme extract solution.

### 3.10. GST Activity Determination Assay

Glutathion *S*-transferase activity was determined by an earlier method with little modifications [36]. Ten microliters of ten-times diluted enzyme extract solution was added to a total volume of 200 μL in microplate wells. The reaction mixture was incubated with 10 mM glutathione at 25 °C for 10 min and added 10 μL 10 mM 1-chloro-2,4-dinitrobenzene (CDNB). Enzyme activity was measured spectrophotometrically at 340 nm using a TECAN™ Infinite^®^ 200 PRO multimode micro-plate reader. The enzyme activity was determined using the extinction coefficient of 9.6 mM^−1^ cm^−1^ for CDNB.

### 3.11. Kinetic Properties of GST

The enzyme extract solution was prepared as mentioned above. Ten microliters of 10 μM cantharidin dissolved in acetone was added to the reaction mixture as an inhibitor. *S*-Hexylglutathione (GTX) in a concentration of 10 μM was used as a positive control. The reaction was started by the addition of 10 mM glutathione and the reaction mixture was incubated at 25 °C for 10 min and finally added 10 μL of 10 mM CDNB. The enzyme activity was measured spectrophotometrically at 340 nm using a TECAN™ Infinite^®^ 200 PRO multimode micro-plate reader. The enzyme activity was determined using the extinction coefficient of 9.6 mM^−1^ cm^−1^ for CDNB.

### 3.12. Kinetic Properties of Purified Soluble rHaGST

In this assay the purified rHaGST as mentioned above was used as the enzyme source. Ten microliters of 10× diluted purified GST enzyme solution was added to the reaction mixture. Ten microliters of 10 μM cantharidin dissolved in acetone was added to the reaction mixture as an inhibitor. *S*-Hexylglutathione (GTX) in a concentration of 10 μM was used as a positive control. The reaction was started by the addition of 10 mM glutathione and the reaction mixture was incubated at 25 °C for 10 min and added 10 μL 10 mM CDNB. Enzyme activity was measured spectrophotometrically at 340 nm using the TECAN™ Infinite^®^ 200 PRO multimode micro-plate reader. The enzyme activity was determined using the extinction coefficient of 9.6 mM^−1^ cm^−1^ for CDNB. The enzyme kinetics module of the SigmaPlot computer package was used to analyze enzyme kinetics data (SigmaPlot, Systat Software, San Jose, CA. USA).

### 3.13. Determination of IC50

To calculate the concentration of inhibitor that inhibits 50% activity of enzyme (IC50), variable concentration of cantharidin dissolved in acetone were added to the mixture containing 170 μL GST buffer, 10 μL enzyme extract and 10 μL of 10 mM GSH solution at 25 °C. No inhibitor was added to the control. The inhibition reaction was carried out at 25 °C. The value of IC50 was calculated by percent inhibitory activity *vs.* concentration of inhibitor using Microsoft Excel 2003.

### 3.14. Real-Time qPCR Analysis of Gene Expression

The real-time qPCR was performed by a BioRad iQ™ 5 cycler. The real-time qPCR reaction was carried out in PCR strips. SYBR Green was used to detect an amplification signal. The reaction mixture consisted of 1 μL 10× diluted cDNA templates, 0.5 μL of 10 μM forward/reverse primers and Maxima™ SYBR Green/ROX qPCR Master Mix (Fermentas, Beijing, China) in a final volume of 25 μL. Forward and reverse primers were used as mentioned above. The real time PCR condition used: initial denaturing at 95 °C for 30 s., forty cycles of 95 °C for 10 s, 60 °C for 30 s and 72 °C for 30 s. The real time data were acquired at 72 °C. Three replicate for each sample were used for real time PCR analysis. The quantification of the relative transcript levels was performed using the comparative CT method. The expression ratio (*R*) was calculated as recommended by the manufacturer and corresponds to 2^−ΔΔCT^, where:

(1) $$\text{Ratio}=\frac{{\left(E_{\text{target}}\right)}^{\Delta C_{\text{T}}\;\text{target }(\text{calibrator}-\text{test})}}{{\left(E_{\text{reference}}\right)}^{\Delta C_{\text{T}}\;\text{target }(\text{calibrator}-\text{test})}}$$

Relative quantification relies on the comparison between expression of a target gene *versus* a reference gene (*Helicoverpa armigera* β-actin) and the expression of the same gene in the target sample *versus* the reference sample [37].

### 3.15. Homology Modeling of HaGST

In this study, we used glutathione *S*-transferase of *Helicoverpa armigera* (HaGST) (ABK40535) obtained from the GenBank database [21] having 220 amino acid residues as a model sequence. All Homology Modeling computations were performed using the Modeller 9.10 [38] on a Linux High performance workstation based on 2 Intel Xeon 5680 processors (Super Micro Computer Inc., San Jose, CA, USA).

Using our model HaGST sequence as a probe, the PDB95 database was searched for non-redundant PDB sequences to select the most appropriate template for our query sequence. In order to visualize differences among 12 similar structures selected at 95% sequence identity as the candidate template, we compared all of the structures in the dendrogram, which can generate the weighted pair-group average clustering based on a distance matrix. The best template was picked according to the crystallographic *R*-factor and overall sequence identity. The sequence alignment between the HaGST sequence and the best template was generated by Align2D and also identified by ClustalX with the Blosum scoring function [39]. The best alignment was selected according to both the alignment score and the reciprocal positions of the conserved residues, especially those in or close to the GTX-binding sites of the template.

Once the target template alignment was constructed, it calculated 100 3D models of the target automatically by the automodel of Modeller using the optimization and refinement protocol. Each model was first optimized with the variable target function method (VTFM) with conjugate gradients (CG), and was then refined using molecular dynamics (MD) with simulated annealing (SA). We also used the loop model class in Modeller to refine the conformation of the loop between residues. To measure the relative stability of the protein conformation, GA341 and Discrete Optimized Protein Energy (DOPE) scores were employed and credible structure of HaGST was selected based on the lowest DOPE energy. MolProbity was used to generate the Protein Main-Chain dihedral Ramachandran map to identify the rationality of the stereochemical for the structure [40].

### 3.16. Molecular Docking Simulations

All the simulations and docking work was performed using the software, GOLD 5.1 [41]. GTX is bound tightly inside the active-site pocket formed by residues Leu6, Ser9, Ala10, Pro11, Leu33, Met34, His38, His50, Ile52, Glu64, Ser65, Arg66, Tyr105, Phe108, Tyr113, Ile116, Phe117, Phe203 and Phe207 as observed from the crystal structure. This pocket was selected as a binding site for cantharidin, so we aligned the 3D model of HaGST to the crystal structure by the superimposition in pymol 1.3r1 edu [42], saved the coordinates of the 3D model of HaGST, then used the coordinates of the CA2 atom in GTX to define a centroid close to the center of the active site with the radius of 10 Å. In order to adopt the best sets of docking parameters and obtain the reliability of the docking results, the GTX was first docked into the binding pocket. The chemical structure of the cantharidin was derived from PubChem [43] and directly used the structure of GTX from 1PN9. Mercury 3.0 software [44] was used to add hydrogen atoms and the coordinates were saved as MOL2 files. Hydrogen atoms were added to the 3D model structure of HaGST and correct ionization and tautomeric states of residues such as Asp, Glu and His were defined, allowing serine, threonine and tyrosine hydroxyl groups, as well as lysine NH^3+^ groups to rotate during docking. All torsion angles in each ligand were allowed to rotate freely. An accurate GTX-GST complex possessing the smallest root-mean-square deviation of the docked conformation of GTX from its conformation in the crystal structure named 1PN9 was obtained first by docking using Goldscore and then by Chemscore after tuning parameters of Gold software several times. Similar docking parameters were adopted for cantharidin and GTX.

### 3.17. Binding Energy Calculations

In order to compare the binding affinity between the cantharidin and GTX with the HaGST model, a ChemScore function was applied to measure the affinity data by a ranking made according to delta value. ChemScore estimates of the total free energy change that occurs on ligand binding were estimated:

(2) $$\Delta G_{binding}=\Delta G_{\text{O}}+\Delta G_{hbond}+\Delta G_{metal}+\Delta G_{lipo}+\Delta G_{rot}$$

## 4. Conclusions

In brief, the results from our investigations revealed that the cantharidin is a potent inhibitor of the GSTs *in vivo* and the HaGST *in vitro*. We therefore suggest the use of cantharidin based bio-insecticides directly or as a synergist where insecticide resistance is related to the over expression of GSTs.

## Figures and Tables

**Table 1:** Delta G values of compounds, GTX and cantharidin against the HaGST 3D model. A ChemScore function was applied to measure affinity data by ranking according to ChemScore delta value.

Molecule	Score	DG	S (hbond)	S (lipo)	H (rot)	DE (clash)	DE (int)
GTX	16.76	−19.65	3.87	142.65	6.04	0.10	2.79
Cantharidin	21.58	−21.86	1.80	88.77	0.00	0.28	0.00

**Table 2:** Oligonucleotide primer sequences for the reverse transcription and the real-time qPCR used in this study.

Name (bp)	Sequence (5-3)	Usage	*T*_m_^a^	Corresponding cDNA region
HaGST-F (29)	CGGATCCATGTCCTTAGACTTGTATTACG	RT	59.2	43–705
HaGST-R (30)	CGAAGCTTTTACAATTCAGTTTTAGCTTTT	RT	56.6	
HaGST-Fq (19)	ACGCTTTACCCAAGATTTG	Real-time qPCR	60	316–430
HaGST-Rq (19)	GGAATGTGTTGAGGAAGTG	Real-time qPCR	60	
HaBA-Fq (18)	GTATTGCTGACCGTATGC	Real-time qPCR	59.7	11–s152
HaBA-Rq (18)	ATCTGTTGGAAGGTGGAG	Real-time qPCR	60	

a Melting temperature (*T*_m_) of the primers was calculated by Beacon Designer 7 software.
